# Antibiotic Resistance Genes in the Subgingival Microbiome in Periodontitis: A Scoping Review of Prevalence, Mobility, and Future Directions

**DOI:** 10.7759/cureus.100685

**Published:** 2026-01-03

**Authors:** Seray Z Ozturk, Bernis Aydin, Emine Cifcibasi

**Affiliations:** 1 Periodontology, Istanbul University Institute of Health Sciences, Istanbul, TUR; 2 Periodontology, Istanbul University Faculty of Dentistry, Istanbul, TUR

**Keywords:** antibiotic resistance genes, antimicrobial resistance, molecular sequencing, oral dysbiosis, periodontal disease (pd), periodontitis, q - pcr, shotgun metagenomic sequencing, subgingival microbiota

## Abstract

The objective of the study is to evaluate the prevalence, diversity, and mobility of antibiotic-resistant species and resistance genes within the subgingival microbiome of patients with periodontitis. A systematic scoping review was conducted in accordance with PRISMA-ScR (Preferred Reporting Items for Systematic Reviews and Meta-Analyses Extension for Scoping Reviews) guidelines. Five electronic databases were searched for studies published between January 2020 and December 2025 that used molecular techniques (shotgun metagenomics, PCR/qPCR, 16S + PCR) to detect antibiotic resistance genes (ARGs) and mobile genetic elements (MGEs) in the subgingival plaque of patients with clinically diagnosed periodontitis. Only peer-reviewed articles presenting original data were included; reviews, animal studies, and investigations lacking clear methodological details were excluded. Data extraction included study design, sample size, identified ARGs, associated MGEs, and clinical context. Nine eligible studies involving over 900 subgingival samples were identified. A core resistome was consistently identified across all cohorts, predominantly comprising tetracycline genes (tetM, tetQ, tet32) and macrolide-lincosamide determinants (ermB, ermF, msrD), as well as β-lactamase genes such as cfxA. Sites affected by periodontitis showed higher abundance of these ARGs than healthy controls. Mobile elements, especially Tn916-family conjugative transposons, were often associated with macrolide resistance genes, suggesting potential for horizontal transfer. Methodological differences prevented meta-analysis, and no study compared results based on the 2017 stage/grade classification of periodontitis. The subgingival resistome in periodontitis features a consistent set of tetracycline, macrolide, and β-lactam resistance genes that are increased in disease and frequently associated with mobile transposons. Currently, the evidence remains primarily descriptive; future research should include standardized antibiotic washout periods, longitudinal follow-up, stage/grade stratification, and integrated multi-omics approaches to evaluate functional activity and guide personalized antimicrobial therapies.

## Introduction and background

Periodontitis is a chronic multifactorial inflammatory disease associated with a dysbiotic plaque biofilm and characterized by progressive destruction of the tooth-supporting apparatus [1]. Clinical signs include attachment loss, alveolar bone resorption, and gingival inflammation.

Dysbiosis is the shift from a balanced microbial community to a state that promotes disease. This change is not merely an increase in pathogenic bacteria but also involves altered microbial behavior in response to environmental triggers. These changes can lead to higher expression of virulence factors, the spread of resistance genes, and structural reorganization of biofilms, all of which support disease persistence [2].

Antimicrobial resistance (AMR) has emerged as a major global health threat. According to the World Health Organization, drug-resistant infections could lead to up to 10 million deaths per year by 2050 [3, 4]. Historically, AMR has been mainly associated with hospital- and gastrointestinal-associated pathogens. Still, recent studies show that the oral cavity, especially the subgingival microbiota, is also an important reservoir of AMR [5, 6]. The oral microbiome contains over 700 microbial species, of which more than 500 are found in the subgingival area [7]. Periodontal pathogens can access the bloodstream or lymphatics, contributing to systemic inflammatory and autoimmune conditions, including cardiovascular disease, Alzheimer’s disease, colorectal cancer, metabolic disorders, and pregnancy complications [8].

Antimicrobial exposure, whether through systemic prescriptions or local periodontal therapies, applies selective pressure on the oral microbiota, enriching resistant strains and expanding the subgingival resistome. The resistome is defined as the complete collection of antibiotic resistance genes (ARGs) within a microbial community [5, 6]. In addition to antimicrobial exposure, dental and orthodontic interventions have been shown to alter oral microbial diversity and community structure, thereby reshaping local ecological pressures that may influence AMR dynamics within the oral microbiome [9]. Although mechanical debridement remains the cornerstone of periodontal therapy, systemic antibiotics may be required in cases with severe tissue breakdown or high-risk profiles. Consensus guidelines from the European Federation of Periodontology (EFP) support targeted antibiotic use under defined clinical conditions [10, 11]. Given this context, the rising prevalence of AMR in the oral microbiome poses a growing challenge for effective periodontal treatment.

Multiple molecular studies have shown that ARGs are widespread in the subgingival microbiome of individuals with periodontitis. Frequently identified β-lactamase-associated ARGs include cfxA and blaTEM [12]; macrolide resistance genes include ermB [13]; and tetracycline resistance genes include tetM and tetQ [12, 14]. These resistance determinants are commonly found in disease-associated taxa such as Prevotella, Fusobacterium, Porphyromonas, and Treponema, as well as in commensal *Streptococcus* species that act as ARG reservoirs [15, 16]. Supporting this, metagenomic studies consistently link periodontitis to increased ARG diversity and abundance, reflecting a significant restructuring of the subgingival resistome [6, 15].

Despite advances in genomic profiling, most current studies remain descriptive and focus only on gene detection rather than functional activity or transcriptional expression [5, 17]. Targeted PCR assays enable sensitive detection of predefined resistance genes [18]. In contrast, shotgun metagenomics provides an untargeted overview of all genetic material within a sample, allowing broader characterization of microbial communities and resistance profiles [19]. This limits our understanding of whether detected ARGs are active, transferable, or clinically relevant. Only a few metatranscriptomic analyses have explored gene expression patterns in periodontal disease, and these studies show that transcriptionally active communities differ markedly from DNA-based taxonomic profiles [20], underscoring the need for integrated multi-omics approaches.

Another critical gap relates to the mobility of ARGs. Mobile genetic elements (MGEs), collectively referred to as the mobilome, include plasmids, insertion sequences (ISs), transposons, and integrative and conjugative elements (ICEs). These elements play a central role in horizontal gene transfer (HGT) among oral bacteria [21]. Genomic reconstructions also suggest that MGEs contribute substantially to the dissemination of resistance and virulence traits in periodontitis-associated communities [22]. These findings collectively indicate that the subgingival biofilm is not merely a passive reservoir of ARGs but a dynamic gene-exchange network capable of accelerating the spread of resistance [23].

Studies that simultaneously investigate the presence, expression, and mobility of ARGs remain rare. For example, while efflux pump genes such as acrAB, mdt, and oqxB are often identified in oral metagenomes, their actual activity and contribution to biofilm-associated resistance are rarely examined [17]. The lack of integrated functional, genomic, and mobilome analyses slows progress toward a more complete mechanistic understanding.

The objective of this scoping review is to synthesize current evidence regarding the presence, expression, and mobility of ARGs within the subgingival microbiome of individuals with periodontitis, while identifying methodological trends, knowledge gaps, and priorities for future research.

## Review

Materials and methods

Study Design 

The scoping review was conducted in accordance with the methodological framework of Arksey and O’Malley [24] and the updated PRISMA-ScR (Preferred Reporting Items for Systematic Reviews and Meta-Analyses Extension for Scoping Reviews) guidelines [25]. The protocol was developed a priori to ensure transparency in the identification, selection, and synthesis of relevant evidence. As this study is a literature-based scoping review, no licensed questionnaires, proprietary measurement instruments, or restricted scoring systems were utilized.

The review aimed to systematically map existing evidence on the presence, expression, and mobility of ARGs within the subgingival microbiome of individuals with periodontitis.

To ensure methodological comparability and reflect the current state of molecular techniques in periodontal resistome research, the literature search was limited to studies published within the last five years (January 2020 to December 2025), given the rapid advancements in sequencing technologies, resistome profiling tools, and mobilome analysis pipelines. Earlier landmark studies were included only for background context in the Introduction and were excluded from data extraction and synthesis.

Eligibility Criteria

Studies were included if they involved human participants diagnosed with periodontitis based on AAP/EFP case definitions or similar clinical criteria, and if they collected subgingival plaque (or subgingival plaque combined with saliva) for analysis. Eligible studies were required to use molecular methods for ARG detection, such as shotgun metagenomics, whole-genome sequencing, PCR/qPCR assays, 16S rRNA sequencing followed by targeted PCR, or specialized resistome or mobilome profiling tools such as CARD, ResFinder, or MobileElementFinder.

Eligible study designs included randomized or controlled clinical trials and observational human research (cross-sectional or case-control). Studies had to report at least one ARG-related outcome, such as ARG prevalence or diversity, MGEs, HGT mechanisms, or gene expression or activity. Only articles published in English were included.

Exclusion criteria included studies relying only on phenotypic susceptibility testing without genetic confirmation, those analyzing only non-subgingival samples (such as saliva or supragingival plaque), in vitro or animal studies, and non-primary publications such as narrative reviews, commentaries, conference papers, or preliminary data. Studies that focused solely on antibiotic consumption or prescription patterns, without microbial analysis, were also excluded. No standardized antibiotic washout period was imposed across included studies; reported exclusion windows varied between studies and were therefore analyzed descriptively.

Search Strategy

A comprehensive search strategy was implemented across the following databases: PubMed/MEDLINE (198 results), Scopus (394 results), Web of Science (44 results), and EBSCOhost (27 results). The search covered January 2020 to December 2025.

The core search terms combined concepts related to periodontitis, subgingival microbiota, ARGs, and molecular detection techniques, using Boolean operators, truncation, and database-specific controlled vocabulary (MeSH). Reference lists of the included studies were manually screened to identify additional eligible publications.

The primary search string was as follows:

(periodontitis OR periodontal disease) AND (subgingival OR plaque OR subgingival biofilm) AND (antibiotic resistance genes OR ARGs OR resistome OR antimicrobial resistance) AND (metagenomics OR shotgun sequencing OR molecular OR PCR OR qPCR OR WGS OR 16S sequencing)

Study Selection

Two independent reviewers (Seray Zeynep Öztürk and Emine Çifcibaşı) screened all records through a two-step process. First, 633 titles and abstracts were assessed for relevance, followed by full-text evaluation of 53 articles. Any disagreements were resolved through discussion, with a third reviewer (Bernis Aydın) providing adjudication when necessary.

**Figure 1:**
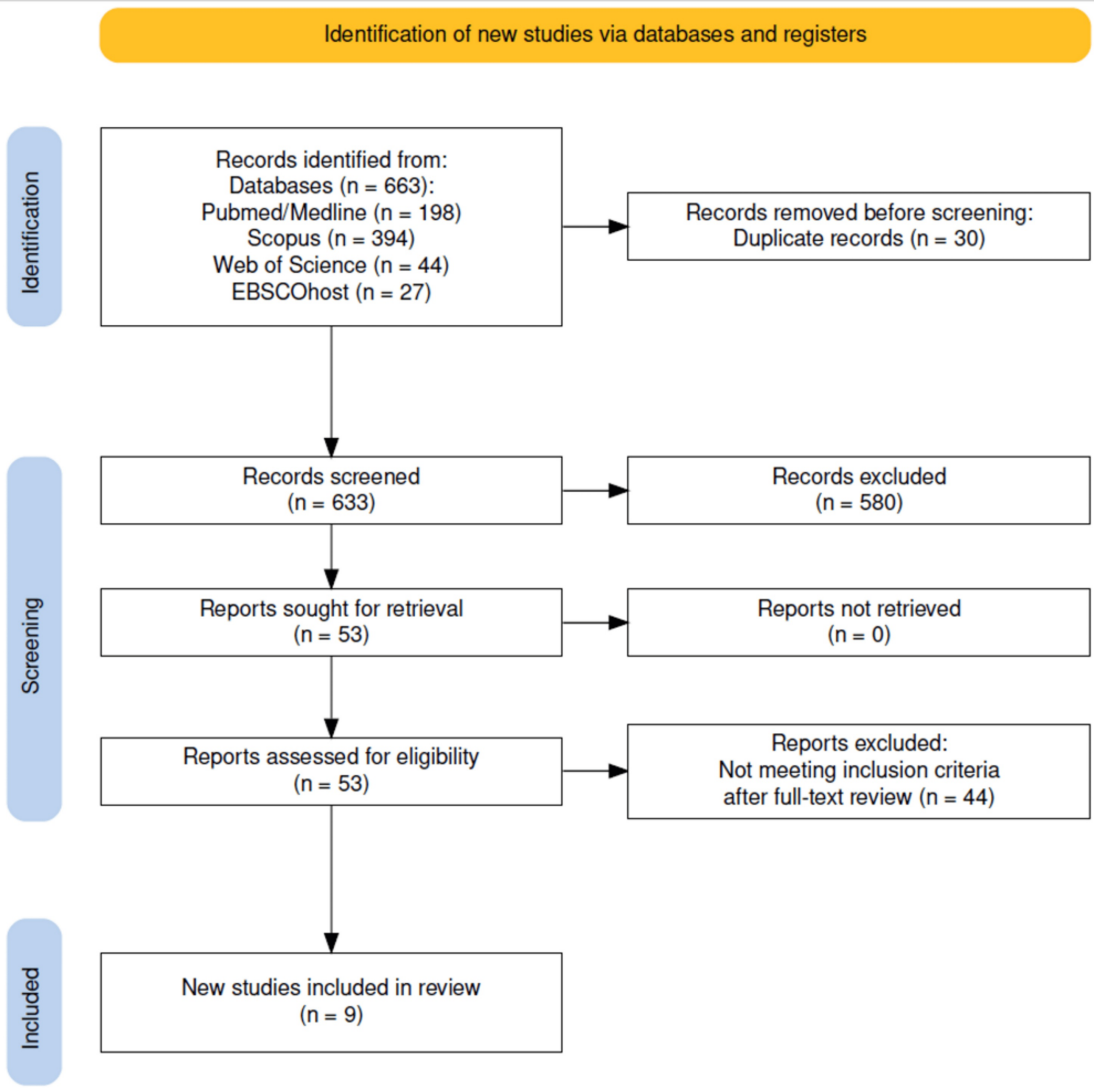
PRISMA Flow Diagram of the Study Selection Process PRISMA flow diagram summarizing the study selection process [25]. PRISMA: Preferred Reporting Items for Systematic Reviews and Meta-Analyses.

A total of 663 records were identified, 30 duplicates were removed, and 633 unique records were screened. After excluding 580 studies at the title/abstract stage, 53 full texts were reviewed; 44 were excluded for reasons such as wrong sample type, absence of molecular detection, non-subgingival sampling, or being review-type articles. Ultimately, nine studies met all eligibility criteria and were included in the final synthesis. The selection process is summarized in Figure 1. A descriptive appraisal of methodological quality, using an adapted Joanna Briggs Institute (JBI) checklist, was conducted to support interpretation of the included evidence. Details are provided in the Appendices.

Data Extraction

Data extraction was performed independently by two reviewers using a standardized form adapted from the JBI scoping review template. Extracted variables included study characteristics (population, setting, sample size), sampling details, health status (healthy vs. periodontitis), molecular methods (shotgun metagenomics, PCR, 16S+PCR), ARG identification tools (CARD, ResFinder, ARG-ANNOT, AMRFinder, SARG, BacMet), detected ARGs, reported MGEs (e.g., Tn916, insertion sequences, integrons), taxonomic carriers of resistance genes, and key conclusions or clinical implications. An overview of the included studies, including study design, sample sources, molecular approaches, and major ARG and MGE findings, is provided in Table 1 [5, 12, 14-16, 23, 26-28].

**Table 1:** Characteristics of Included Studies (2020–2025) ARGs: antibiotic resistance genes; MGEs: mobile genetic elements; PCR: polymerase chain reaction; CARD: Comprehensive Antibiotic Resistance Database.

Author (Year)	Country	Study Design	Sample	Molecular Methods	Main ARGs	MGE Notes
Zhang et al., 2025 [14]	China	Cross-sectional	Subgingival plaque (n: 21, n:84 subgingival plaque samples)	16S rRNA V3–V4 amplicon sequencing (Illumina), Shotgun metagenomics (CARD), Strain isolation + 16S Sanger; tetM PCR & RT-qPCR	tetM, cfxA, ermF	tetM (Tn916/Tn1545 family)
Anderson et al., 2023 [5]	Germany	Cross-sectional	Subgingival Plaque+ saliva (n:180) (63 healthy, 55 periodontitis)	Shotgun metagenomics NovaSeq 6000, 2x150 bp) ARG identification via ABRicate (Res Finder, CARD, NCBI, ARG-ANNOT); anaerobic culture MALDI-TOF, PCR	msrD, mefA, cfxA, ermB/ermF, tetM/tetQ/tet32, pgpB	Tetm, ermF/ermB, mefA, msrD (Tn916/Tn1545 family)
Gager et al., 2023 [12]	Germany	Cross-sectional	Subgingival plaque (n=39 patients, 124 subgingival plaque samples)	Shotgun metagenomics (CARD)	cfxA, cfxA3, blaTEM, tetM, ermB	Suggested MGE linkage; not mapped
Romero-Martinez et al., 2023 [28]	Spain	Laboratory based molecular characterization of clinical isolates	Subgingival Plaque (n:8) (2 periodontitis, 1 peri-implant mucositis, 5 peri-implantitis	PCR (16S rRNA, pilN, ftxA), Whole-genome sequencing (Illumina NovaSeq 6000), ARG detection using AMRFinderPlus, MGE analysis (BLAST, ORFfinder)	Tet32, ermB	Tet32 (transposons), ermB (Tn916/Tn1545 family)
Sparbrod et al., 2022 [23]	Germany	Cross-sectional	Subgingival Plaque (n: 19)	Shotgun metagenomics Illumina NextSeq 500 ARG identification via ResFinder; anaerobic culture MALDI-TOF-MS, 16s rRNA sequencing for uncultivable isolates	ermF/ermB/ermG/mefA, cfxA/cfxA3/cfxA4/cfxA5, tet32/tetM/tetQ/tetO	ermF/ermB, tetM/tet32 (Tn916/Tn1545 family)
Kang et al., 2021 [15]	China	Cross-sectional	Subgingival plaque (pre/post SRP) n: 112 subgingival plaque samples	Shotgun metagenomics (Illumina), ARG identification SARG (ARGs-OAP pipeline), CARD, BacMet (MRGs), MGE database (Tn916, transposases)	ermB, tet32/tetM/tetQ/tetW, cfxA, mefA, PBP- 1A/PBP- 2X, emrA/emrB/mdtG	Tn916/Tn1545 (orf9/15/16/17), IS91, transposases (tnpA)
Almeida et al., 2020 [16]	Brazil	Cross-sectional	Subgingival plaque+ saliva (n:110), (25 healthy, 61 gingivitis, 24 periodontitis)	16S rRNA gene sequencing and targeted PCR assays.	Erm, pbp2b, aac(6’), mecA, blaTEM, tetM	Erm (Tn916/Tn1545 family)
Arredondo et al., 2020 [26]	Spain	Cross-sectional	Subgingival plaque (n: 259)	Culture-based isolation; 16S rRNA PCR + Sanger; multiplex PCR for tet genes, erm(B), and intTn (Tn916).	tetM/tet32/tetO/ tetW/ tetL/ tetQ/ tetB, ermB	Tn916/Tn1545 transposons (intTn), often co-carry tet(M) and erm(B).
Arredondo et al., 2020 [27]	Spain	Cross-sectional	Subgingival plaque (n:24) (12 periodontitis, 12 healthy patients)	16S rRNA sequencing, PCR	TetQ/tetM/tet32, ermF	Not directly assessed; ARGs likely associated with conjugative transposons (tetQ/tetM)

Data Synthesis

Due to the methodological differences among the included studies, a descriptive synthesis was performed instead of a meta-analysis. Studies were categorized based on (i) molecular methodology (metagenomics versus PCR-based), (ii) participant health condition (healthy vs periodontitis), (iii) ARG categories (such as tetracycline, macrolide, β-lactam, multidrug efflux), and (iv) reported MGEs. Key themes were identified to aid interpretation and inform the discussion and future research directions.

Results

A total of nine studies published between 2020 and 2025 met the inclusion criteria for this scoping review. All studies investigated ARGs within subgingival plaque using molecular approaches, including shotgun metagenomics, 16S amplicon sequencing combined with PCR-based ARG detection, and multiplex PCR assays. The characteristics of all included studies are summarized in Table 1.

Study Characteristics

The studies were conducted across five countries (Germany, China, Spain, Brazil, India), reflecting broad international interest in the molecular characterization of antibiotic resistance within subgingival microbial communities. Most studies employed a cross-sectional design, including laboratory-based molecular characterization of clinical isolates, and sample sizes ranged from 8 to 259 individuals, with total subgingival plaque samples per study ranging from 8 to 259. Collectively, these studies represent molecular data from >900 subgingival plaque samples.

Molecular Approaches Used

Shotgun metagenomics was the most frequent molecular strategy, applied in five studies [5, 12, 14, 15, 23]. These studies used major resistome databases, including CARD, ResFinder, ARG-ANNOT, NCBI AMRFinder, SARG/ARGs-OAP, and BacMet.

Three studies employed PCR-based approaches [5, 14, 16, 26-28], either as the primary identification method or in combination with sequencing. These PCR-focused studies, by the nature of the technique, targeted only predefined resistance determinants (tet gene family), macrolide resistance genes (erm variants), and integrase genes (e.g., intTn), often followed by sequencing for taxonomic confirmation. Two studies [15, 26] additionally screened MGEs through dedicated integrase and transposase databases.

Distribution of ARGs

Despite methodological heterogeneity, the included studies consistently identified a core set of ARGs in subgingival plaque. Across all datasets, the most frequently reported genes belonged to the tetracycline resistance family, including tetM, tetQ, tet32, tetW, tetO, tetL, and tetB [5, 12, 14, 15, 23, 26-28]. Among these, tetM appeared as the most consistently detected determinant across both metagenomic and PCR-based studies.

Macrolide-lincosamide-streptogramin (MLS) resistance genes, including ermB, ermF, ermR, mefA, and msrD, were also frequently reported [5, 12, 15, 16, 23, 26-28].

β-lactam resistance genes were primarily represented by the cfxA gene family, including variants cfxA3, cfxA4, and cfxA5, which were recurrently identified in five metagenomic studies [5, 12, 15, 23]. Additional β-lactam-associated determinants, such as blaTEM, were detected in both culture-PCR and shotgun datasets [12, 16].

Overall, the collective dataset indicates that tetracycline, macrolide, and β-lactam resistance determinants dominate the subgingival resistome, regardless of geographic location or sequencing platform.

Mobile Genetic Elements (MGEs)

Eight of the nine studies reported findings related to MGEs, though the depth of analysis varied significantly. The Tn916/Tn1545 family of conjugative transposons was the most frequently associated MGE group, with studies reporting either the presence of integrase genes (intTn) or co-localization of tet, erm, mef, and erythromycin genes with Tn916/Tn1545-like elements [5, 14-16, 23, 26-28].

Kang et al. provided the most extensive mobilome profile, identifying Tn916-associated open reading frames, IS91-family insertion sequences, transposases, and additional signatures of plasmid-associated integrases [14]. These data represent the deepest MGE characterization among available subgingival studies. Gager et al. did not perform explicit MGE mapping, though ARG profiles suggested potential linkage to conjugative elements [12].

Discussion

Subgingival Microbiome as a Structured Reservoir of AMR

Across the nine molecular investigations, a clear picture emerges: whether the site is healthy or diseased, the subgingival microbiota behaves as a stable, organised reservoir of antimicrobial-resistance genes (ARGs). This agrees with early observations that oral biofilms contain dense networks of transferable DNA [8, 29]. More recent molecular investigations have taken that idea a step further, showing that the “oral resistome” is not a static collection of genes but a dynamic pool that reshapes itself in response to antibiotic pressure, metabolic stress, and the structural changes that accompany disease [30]. The selective pressures imposed by inflammation, anaerobiosis, and high microbial density appear to shape which ARGs are maintained and amplified, in line with recent ecological AMR frameworks [31]. In addition to host- and disease-related pressures, dental materials used in clinical practice may exert local antimicrobial effects, creating micro-environmental selective pressures that can influence oral microbial composition and potentially shape AMR dynamics [32].

Across the molecular studies we surveyed, both shotgun metagenomics and targeted PCR panels show a clear pattern. ARGs are almost ubiquitous in the oral cavity, yet their abundance, organization, and mobility differ sharply between health and periodontitis. Almeida and colleagues, for example, reported that more than 70% of oral samples carried at least one ARG, with macrolide-resistance genes such as erm often residing on Tn916-family conjugative transposons [16]. Anderson and co-workers extended these findings in a cohort of 179 individuals, coupling metagenomic profiling with phenotypic susceptibility testing. They showed that total ARG richness was higher in the healthy and caries groups, but that periodontitis patients harboured a distinct “resistotype” enriched for msrD, mefA, ermF, tet32, tetQ, and the *Porphyromonas gingivalis*-associated gene gpgb [5]. This shift mirrors the dysbiotic ecotypes that define advanced disease, suggesting that the resistome is reorganised at the functional-gene level as inflammation deepens.

Healthy sites consistently carried a core resistome dominated by tetracycline and macrolide-lincosamide determinants, but at lower relative abundance than diseased pockets. In metagenomic datasets, periodontitis was associated with selective enrichment of tetM, tetQ, tet32, ermF, ermB, and cfxA-like β-lactamases [5, 12, 14, 15]. PCR-based studies of subgingival isolates reported the same “signature” genes, with tetQ and ermF particularly prevalent in anaerobic rods from diseased sites [16, 21, 25]. Together, these findings indicate that AMR in the subgingival environment is not incidental but represents a reproducible, disease-modulated resistome architecture.

Health-Disease Resistome Dynamics

Despite substantial overlap in the types of ARGs detected in health and disease, almost all studies identified quantitative shifts in ARG abundance associated with periodontitis. In PCR-based studies, tetQ, tetM, and tet32 were more frequent among isolates from patients with periodontitis than among controls, and ermF (occasionally with ermB or mefA) was enriched in disease-associated anaerobes [12, 27].

Shotgun metagenomic studies extend these observations to the community level. Deep periodontal pockets contain larger proportions of tetracycline and macrolide resistance genes, together with more β-lactamases and multidrug efflux systems [5, 12, 15]. Some studies also reported similar enrichment in periodontitis, with partial normalization following scaling and root planing. Zhang et al. [14] further demonstrated that recent antibiotic exposure selectively enriched streptococci carrying tetracycline and macrolide determinants in periodontitis plaque. Collectively, these findings support a model in which dysbiosis reorganizes the resistome quantitatively rather than introducing entirely new ARG families, consistent with broader oral AMR reviews [8, 33].

Notably, none of the nine studies stratified resistome data according to the 2017 staging and grading framework, despite recruiting patients with moderate to advanced disease [e.g., 27]. This limits conclusions about whether more destructive or rapidly progressing periodontitis presents distinct resistance profiles. This gap has also been noted in recent reviews of AMR-periodontitis [31].

Taxonomic Carriers: Commensals as the Primary ARG Reservoir

A striking and consistent observation is that commensal and opportunistic taxa, rather than classical red-complex pathogens, act as the principal ARG carriers. In both PCR-based and metagenomic studies, *Prevotella*, *Fusobacterium*, *Parvimonas*, *Veillonella*, *Gemella*, and various oral streptococci repeatedly harboured tet, erm, and cfxA-like genes [5, 14, 16, 27]. Whole-genome sequencing of *Filifactor alocis* isolates from periodontal and peri-implant lesions additionally revealed tet(32) and erm(B) embedded in mobile regions, confirming that highly virulent but previously under-characterised species can also serve as carriers [28].

Moreover, it has been speculated that *Porphyromonas gingivalis* and *Tannerella forsythia*, as key disease drivers, are rarely major ARG hosts, underscoring the idea that flexible commensals serve as the genetic “bridge” for resistance dissemination [8, 29, 31].

Contributions of Metagenomics vs PCR-Based Evidence

Shotgun metagenomic studies [5, 12, 14, 15] uncover a broad resistome, revealing ARG classes missed by targeted PCR, such as multidrug efflux pumps, aminoglycoside‑modifying enzymes, and multiple β‑lactamase subtypes. These approaches also enabled quantitative comparisons of ARG abundance between health and disease and, in the case of Kang et al. [15], before and after non-surgical periodontal therapy.

In contrast, PCR-based studies [16, 27] demonstrated high specificity for clinically relevant determinants, including tet(Q), tet(M), tet(32), erm(F), erm(B), and selected β-lactamase genes. Arredondo et al. [25] demonstrated that subgingival isolates from periodontitis patients carried an array of β-lactamases, including bla_TEM, bla_SHV, bla_CTXM, cfxA, cepA, cblA, and ampC, highlighting the breadth of β-lactam resistance potential in this niche. Both Almeida et al. [16], Sparbrod et al. [23], and Arredondo et al. [27] combined PCR with phenotypic susceptibility testing.

Sparbrod et al. [23] reported only partial concordance between detected tetracycline and β-lactam genes and clinical resistance phenotypes, reinforcing AMR review observations that gene presence does not guarantee expression or phenotypic resistance, which is strongly modulated by regulatory, metabolic, and ecological factors [31]. Overall, the metagenomic and PCR-based evidence are complementary: metagenomics outlines the scope and relative abundance of the resistome, while culture and PCR-based approaches link those findings to functional outcomes.

Mobilome Analyses and Transfer Potential

Only a few studies examined the genomic context of ARGs, but the evidence strongly suggests that conjugative transposons and other MGEs are the main drivers of the observed ARG structure. Kang et al. [15] identified diverse insertion sequences, transposases, and integrase-containing regions enriched in periodontitis-associated communities, indicating an expanded mobile gene pool in dysbiotic pockets. Whole-genome sequencing of *F. Alocis* showed that tet(32) and erm(B)were located adjacent to transposase and recombinase genes, consistent with carriage on Tn916-like conjugative elements [28].

Although other included studies did not specifically reconstruct MGE architecture, many of the repeatedly observed determinants, such as tet(M), tet(Q), and erm(B), are well-known components of Tn916/Tn1545 family transposons and related elements, extensively described in oral streptococci and other mucosal bacteria [29, 30, 34, 35]. The co-occurrence of tetracycline and macrolide genes within the same isolates or communities [16, 23, 27, 28] further supports a model where linked MGEs facilitate co-selection, meaning that exposure to one antibiotic class may increase resistance to others.

Functional Interpretation and Genotype-Phenotype Discrepancy

Across both PCR- and sequencing-based studies, genotype-phenotype mismatch emerged as a recurring theme. Despite frequent detection of tet and erm genes, not all isolates or communities exhibited corresponding high-level resistance in phenotypic assays [16, 27, 28]. This is consistent with the broader AMR literature, which shows that resistance expression is shaped by transcriptional regulation, metabolic burden, community interactions, and environmental gradients rather than by gene presence alone [31].

The subgingival microbiota is characterized by low oxygen levels, protein-rich substrates, and chronic inflammation, which ARGs may provide ecological fitness advantages even without direct antibiotic pressure, for example, by supporting survival under oxidative stress or facilitating co-selection on mobile elements. These findings support the idea that AMR in periodontitis results from an evolutionary strategy embedded in dysbiosis, rather than just a consequence of exogenous antimicrobial use.

Methodological Heterogeneity and Limitations 

A key methodological challenge in the included molecular studies is the inconsistent criteria for excluding patients with prior antibiotic use, which limits the comparability of baseline resistome profiles. Reported exclusion periods varied considerably, ranging from 6 months [5] to 3 months [23, 26-28], and to 30 days [16]. In contrast, several metagenomic studies did not specify any exclusion window for antibiotic use [12, 15, 28]. 

Systemic antibiotic exposure rapidly selects for and amplifies AMR genes (ARGs) within the oral biofilm. The lack of methodological consensus compromises the validity of quantitative comparisons of ARG prevalence and abundance across study cohorts [15, 36]. This heterogeneity in defining "antibiotic-naive" baselines represents a significant limitation that hinders robust evidence synthesis regarding the disease-modulated subgingival resistome. Future research should use standardized and stringent washout periods to support reliable comparative analyses.

Integrated Synthesis Across All Evidence

Synthesis of the nine molecular studies yields a unified model: periodontitis does not simply increase the number of ARGs but reorganizes the subgingival resistome toward mobile, clinically relevant determinants carried predominantly by anaerobic commensals. This model closely mirrors macro-level insights from recent oral resistome reviews, which emphasize commensals as central ARG reservoirs and highlight the need to integrate AMR ecology into periodontal disease frameworks [8, 31, 33].

Future Directions

The comparative analysis presented in this review highlights the weakest areas of the evidence base and identifies priorities for future research.

First, mobilome-level resolution remains insufficient. Only Kang et al. [15] conducted a dedicated mobilome analysis, identifying insertion sequences, transposases, and Tn916-associated ORFs. Others reported integrase genes [16, 28] or speculated on Tn916 linkages based on known associations [5, 23], but did not confirm genomic context. Future research should employ long-read and hybrid sequencing, dedicated MGE detection pipelines, and strain-level assembly to map ARG-MGE co-localization and quantify which resistance determinants are truly transferable within dysbiotic pockets.

Second, none of the included studies stratified patients according to the 2017 Periodontitis Stage/Grade Classification. Participants were generally categorized as “healthy” or “periodontitis,” without distinguishing between Stage II, Stage III, or Stage IV disease, or accounting for Grade B versus Grade C risk profiles, and only a few reported detailed clinical attachment loss or radiographic parameters alongside resistome data [27]. Larger, well-phenotyped cohorts that integrate staging, grading, and systemic risk factors into resistome analysis are needed to determine whether distinct AMR profiles characterize aggressive versus slowly progressing disease.

Third, functional expression and genotype-phenotype relationships are poorly resolved. Although several studies reported rich tetracycline, macrolide, and β-lactam ARG repertoires [5, 12, 14-16, 26], only a subset systematically compared these with MIC or inhibition-zone data [26], and none assessed ARG expression at the RNA or protein level. Metatranscriptomic, metaproteomic, and functional validation studies will be essential to discriminate silent genetic potential from clinically meaningful resistance.

Fourth, geographical coverage and sample size are limited. The available studies originate from a small number of countries and are largely single-center, with modest sample sizes [12, 14-16, 27]. Multi-center, multi-ethnic cohorts using harmonized sampling and bioinformatic pipelines to capture how regional antibiotic use and host factors shape the global subgingival resistome.

Fifth, longitudinal and treatment-response data are scarce. Only one study examined changes in the resistome following nonsurgical periodontal therapy [15], and none evaluated the impact of systemic or locally delivered antibiotics on ARG and MGE dynamics in periodontal pockets. Prospective longitudinal studies and carefully designed interventional trials are needed to determine whether current treatment strategies reduce, shift, or inadvertently select for particular ARG-MGE combinations. 

Sixth, a major methodological challenge in the included molecular studies is the inconsistent criteria for excluding patients with prior antibiotic use, which limits the comparability of baseline resistome profiles. Future research should focus on dedicated pharmacokinetic and ecological studies to define the optimal, standardized duration for resistome stability after antibiotic cessation.

Finally, translation into periodontal clinical practice is at an early stage. Although this review shows that dysbiotic pockets are enriched for specific mobile resistance signatures, these patterns are not yet incorporated into empirical prescribing or risk-stratification frameworks. Future work should test whether subgingival resistome and mobilome profiling can lead to personalized antimicrobial strategies, monitor high-risk patients, and predict long-term periodontal prognosis, as proposed in recent conceptual reviews [8, 31, 33].

Such approaches will allow the field to determine not only which ARGs are present, but also which are active, transferable, and clinically significant, and how these evolve across the spectrum from health to periodontitis.

## Conclusions

This scoping review shows that the subgingival microbiome is a stable and organized reservoir of ARGs. Dysbiotic periodontal pockets are associated with increased prevalence of tetracycline, macrolide, and β-lactam resistance genes. However, there are inconsistencies in study design, sampling strategies, sequencing depth, and detection methods for ARGs and MGEs. These differences make it complicated to compare results directly and limit the strength of clinical conclusions from meta-analyses. Future studies integrating multi-omics and standardized methods are needed to generate more consistent data and facilitate the translation of genetic findings into personalized care for individuals with periodontitis.

## Appendices

**Table 2:** Overview of Methodological Quality of Included Studies (Adapted JBI Criteria) A descriptive appraisal of methodological quality was conducted using an adapted Joanna Briggs Institute (JBI) checklist. The appraisal focused on population definition, clinical sampling, molecular methodology, outcome reporting, and acknowledgment of confounding factors, and was intended to support interpretation of the evidence rather than to exclude studies based on quality.

Study	Population Defined	Clinical Context and Sampling	Molecular Methods	Outcome Definition	Confounders Acknowledged	Overall Concern
Zhang et al., 2025 [14]	Yes	Yes	Yes	Yes	Unclear	Low
Anderson et al., 2023 [5]	Yes	Yes	Yes	Yes	Unclear	Low
Gager et al., 2023 [12]	Yes	Yes	Yes	Yes	No	Moderate
Romero-Martinez et al., 2023 [28]	Yes	Yes	Yes	Yes	Unclear	Low
Sparbrod et al., 2022 [23]	Yes	Yes	Yes	Yes	Unclear	Low
Kang et al., 2021 [15]	Yes	Yes	Yes	Yes	Yes	Low
Almeida et al., 2020 [16]	Yes	Yes	Yes	Yes	Unclear	Low
Arredondo et al., 2020 [26]	Yes	Yes	Yes	Yes	Unclear	Low
Arredondo et al., 2020 [27]	Yes	Yes	Yes	Yes	Unclear	Low

**Table 3:** Glossary of Key Technical Terms and Abbreviations This table provides concise definitions of key technical terms and abbreviations used throughout the manuscript to aid reader comprehension.

Term/ Abbreviation	Definition
ARG	Antibiotic resistance gene
Resistome	The complete collection of antibiotic resistance genes present in a microbiome
Mobilome	The set of mobile genetic elements capable of horizontal gene transfer
MGE	Mobile genetic element, including plasmids, transposons, insertion sequences, and integrative conjugative elements
HGT	Horizontal gene transfer between microorganisms
Tn916/Tn1545	A conjugative transposon frequently associated with tetracycline resistance genes such as *tet(M)*
ICE	Integrative and conjugative element
Shotgun metagenomics	Untargeted sequencing of total microbial DNA from a sample
CARD	Comprehensive Antibiotic Resistance Database used for ARG annotation
PCR / qPCR	Polymerase chain reaction / quantitative polymerase chain reaction for targeted gene detection
WGS	Whole-genome sequencing
IS	Insertion sequence, a simple transposable element
